# Improving the safety of rapid tranquilisation in older people

**DOI:** 10.1192/bjo.2021.129

**Published:** 2021-06-18

**Authors:** Richard Harris, Rollo Sheldon, Jane McNulty, Scott Cherry

**Affiliations:** Sussex Partnership NHS Trust

## Abstract

**Aims:**

To identify intramuscular rapid tranquilisation (IMRT) events in all >65 years inpatients in Sussex Partnership NHS Foundation Trust (SPFT) and to establish whether accompanying documentation meets SPFT guidelines. This is a re-audit, initial data were collected in 2016. Multimodal intervention has been implemented since initial data collection. In psychiatric inpatients IMRT should be administered as a last resort to calm acutely disturbed patients after verbal de-escalation and an offer of oral medication has failed. IMRT can cause physical health complications and impact therapeutic relationships. Quality improvements made since initial data collection were: an IMRT treatment algorithm for >65s, a teaching package for staff, IMRT prescription area on medicine cards and post IMRT physical monitoring forms – in line with updates to trust IMRT policy.

**Method:**

Retrospective case note audit cycle of 119 patients. Electronic and paper records were reviewed for inpatients >65 years on 1/9/2019. Records were examined for instances of IMRT– the following features were noted: diagnosis; verbal de-escalation; oral medication offered prior to IMRT; IMRT prescription location; and post-IMRT monitoring. Descriptive statistics were performed. This audit was approved by the trust audit committee.

**Result:**

There were 34 RT events in 17 patients, reduced from 83 RT events in 20 patients in 2016. De-escalation was attempted in 62% versus 34% in 2016, oral medication offered first in 71% versus 59% in 2016. Physical monitoring was fully completed in 50% of instances in 2019, an improvement from 23% in 2016.

**Conclusion:**

Education, a new treatment algorithm, medicine card changes, and IMRT physical monitoring forms have improved adherence to trust standards. There was a 49% reduction in IMRT events in 2019 versus 2016. De-escalation is being performed more frequently, and oral sedation offered in more cases. The physical monitoring of patients has improved.

